# Is common sense all you need? Using expert defined rules to identify vulnerability patches instead of machine learning

**DOI:** 10.1007/s10664-026-10860-0

**Published:** 2026-05-07

**Authors:** Aurora Papotti, Serena Elisa Ponta, Antonino Sabetta, Fabio Massacci

**Affiliations:** 1 VUAmsterdam, Amsterdam, Netherlands; 2 SAP, Amsterdam, Netherlands; 3 UTrento, VUAmsterdam, Amsterdam, Netherlands

**Keywords:** First keyword, Second keyword, More

## Abstract

****Goal**:**

Machine learning (ML) has been proposed to identify security fixing commits with mixed success. We evaluate a different alternative in which expert-defined common sense rules with power-law weights are used to identify security fixing commit.

****Experiments**:**

We first evaluated how those rules perform against ML models trained on the same features on which rules are based on the ProjectKB real world dataset of Java security commits. We then ran a think aloud protocol with seven senior analysts to analyze whether the selected rules are consistent with usage. Lastly, we ran the experiment with Master students ($n=90$) to check whether the last ranking or the actual mention of which rules were applicable is beneficial to users with less experience. We found that the common-sense rules defined by security experts have a similar performance to classical ML approaches.

****Findings**:**

Using Shap values to explain earned features, we find that ML models have the same chance corrected accuracy of expert defined rules, and they learned essentially the same top features identified by the experts and only differs on minor features (either among themselves and between the expert features). We observed that the juniors performance are comparable to the experts’ one (CI [0.62, 0.76]). When asked to junior analysts to identify the fixing commits (in the top 10 selected by the tool) showing them which features was responsible for the selection do not seem to help (when compared with a control group with no support). A conclusion of our study is that one might just use ML to first analyze the data and then distill common sense rules that are still effective to apply. More experiments are needed to make features useful for the final decision.

## Introduction

Detailed, code-level vulnerability data are crucial for powering software composition analysis tools (Imtiaz et al. 2021), and support developers to identify known vulnerabilities in open source software dependencies (Ladisa et al. 2023). A key practical question is whether the code we use is affected by a security commit (Pashchenko et al. 2020a; Cabrera Lozoya et al. 2021). If it is not (e.g. our version includes code after the security fixing commit), there is no need to worry; if it is affected, we need to update or understand what the commit does and where it is, as we might be in an unsupported branch.

Once the vulnerability patch is found, automated technologies such as SZZ algorithms have been adapted (Nguyen et al. 2016) to identify commits that likely introduced the bugs (i.e. the vulnerability inducing commits) and are continuously updated (Bao et al. 2022). Several state-of-the-art datasets used in machine learning for vulnerability detection such BigVul (Fan et al. 2020), CrossVul (Nikitopoulos et al. 2021), CVEfixes (Bhandari et al. 2021) all exploit the knowledge of a vulnerability patch to automatically generate labels for training (before the commit the code is vulnerable and after the commit the code is not vulnerable).

Yet, all those algorithms assume that the fixing commit is known. Mapping vulnerability advisories to their fixing commits in the source code is still a challenge (Tan et al. 2021). Hogan et al. (2019) reported that manual labeling is a high-skill, time-consuming task and can still be error prone due to lack of knowledge. For a developer finding the vulnerability patch without automated support is thus a daunting task as the fixing commit might hundreds or thousands of commits from the current point in the branch of the software actually in use (Dashevskyi et al. 2018).

To address this problem, several approaches have been proposed to solve the security commit finding problem by ignoring the semantic of the code and rather retrieve contextual information from the vulnerable repository, from third party vulnerability datasets, issue trackers and other sources. For example, Tracer implements the advisory references crawling technique (Xu et al. 2022), Prospector (Sabetta et al. 2024) uses expert-defined heuristics, PatchScout, leverages machine learning (ML), natural language process (NPL) and pattern matching (Tan et al. 2021) and Hermes extends PatchScout by looking up the bug tracking ID in alternative sources (Nguyen-Truong et al. 2022). Recently, Wang et al. (2022) proposed a novel ranking-based: *VC-MATCH* (Vulnerability-Commit Match). The tool also applies three classification models (i.e. XGBoost, LightGBM, CNN) and uses a voting-based rank fusion method to combine the results of the three models to generate a better result.

Eventually, these technologies are only useful if they finally support the developer in what surveys (Kula et al. 2018) and interviews  (Pashchenko et al. 2020b) have identified as the ultimate problem: how effective it is to select the patch candidate that is *actually* a security patch for a specific vulnerability among the few surviving the mathematical analysis. Existing studies such as PatchScout (Tan et al. 2021), VCMatch (Wang et al. 2022), or Hermes (Nguyen-Truong et al. 2022) have mainly focused on evaluating algorithmic perfomances through quantitative metrics such as precision and recall. However, none of these studies has performed a human-centered validation to assess whether the information provided by the tool effectively supports experts and less experienced users in the “last mile” decision of selecting a fixing commit. As we combine quantitative comparisons with qualitative insights from experts and controlled experiments with junior analysts, our study extends the state-of-the-art from pure algorithmic benchmarking to an empirical evaluation of usability and cognitive alignment between automated reasoning and human judgment. Therefore, the novelty of our work lies in showing how expert-defined rules align with human decision processes and how such alignment compares to machine learning-based solutions.

In this respect, the industrial tool Prospector offers an interesting opportunity for empirical research. At first, it has been released open source, so it can be used for independent research. Secondly, and perhaps more interestingly from our perspective, it is a tool that is *not* based on ML techniques (the latter have also been reportedly used by the same company Cabrera Lozoya et al., 2021): it employs a *set of common sense heuristics* that mimics the strategies inspired by those employed by human security experts.

### Our Contribution

Our main contribution is conducting a systematic human evaluation of how analysts interact with an automated tool to identify the fixing commits. As preliminary study, we first evaluate whether Prospector is not under performing compared to alternative solutions to ML techniques to identify security patches; so we make sure that the human participants do not fail just because the tool is under performing. To the best of our knowledge we did not find any other tool based on expert rules, but only tools based on machine learning models. This is the reason why we think that our study is relevant and brings a new contribution to the research.

As part of this control study we compared Prospector performance with four different traditional ML techniques: (1) linear regression, (2) logistic regression, (3) XGBoost, and (4) LightGBM. We chose XGBoost and LightGBM as they are two of the machine learning models implemented in VCMATCH (Wang et al. 2022). This analysis was performed using industrial ProjectKB dataset (Ponta et al. 2019). We decided to use this dataset to be consistent with the evaluation made by Sabetta et al. (2024). We compare the final aggregate value using chance corrected accuracy (Doswell et al. 1990), an adjusted accuracy metrics used in weather forecasts to account for the bias given by tradional metrics in presence of rare events (a security commit is extremely rare among the thousands to be analyzed). As Prospector implements expert-defined rules on selected features based on heuristics we used the Shapley values of the resulting ML predictions to extract “equivalent weights” assigned by the ML and compare those values against the expert-defined original value. We show that the ML models re-discovered the main features that experts already knew.

The second goal of our study is to understand whether the expert-defined rules are supportive in this final step that we name ‘last-mile’, where the human make the final decision. Noller et al. (2022) have already demonstrated that developers can manage only a handful of rules. We conducted a think-aloud experiment with seven senior security experts from different software companies (with different roles) to explore this aspect of our research problem. Expert-defined rules should be more effective for junior users, as they provide the direct explanation of the prediction. Thus we performed a controlled experiment with Masters’ students. The experiment design is based on three different treatments (groups) and we analyzed whether there was any statistical difference between the groups. In addition, in a validation follow-up experiment with a subset of participants, we also investigated what would happen if we showed the fully ecologically valid report created by Prospector for a specific vulnerability and how far they would have successfully identified commits with a confidence interval 95% using the Wilson method.

### Paper Overview

In Section 2 we present the background of our study, and related works; Section 3 is a brief description of Prospector, for further information please refer to the study by Sabetta et al. (2024). In Section 4 we introduce our research questions and detail our experimental design and in Sections 5 6 7 we respectively report the findings for each research question formulated. Finally, in Section 8 we describe the threats to our study and we conclude with some implications for future works in Section 9.

## Background and Related Works

Traditionally, the process of identifying security fixes, essential to maintaining the security and integrity of software systems, has relied heavily on manual analysis by security experts. This approach is time-consuming, error-prone, and difficult to scale. The growing dependence on open source software (OSS) has further amplified the need for automated tools capable of accurately detecting security-relevant changes or fixes in large codebases.

### Gathering Labeled Data

Various machine learning approaches have been proposed to automate the identification of security-fixing commits (Xu et al. 2022; Tan et al. 2021; Nguyen-Truong et al. 2022). These methods rely on models trained on large datasets that contain both vulnerable and fixed code to learn patterns associated with security vulnerabilities. However, ML-based approaches are often computationally expensive and require substantial amounts of labeled data. In most existing security vulnerability (SV) datasets, vulnerable artifacts (such as functions or lines of code) are labeled based on Vulnerability-Fixing Commits (VFCs) (Le et al. 2024, 2019). This method of data collection is instrumental in building realistic SV prediction models that can perform effectively in practical environments. VFCs are typically documented alongside SVs in security advisories, such as those in the National Vulnerability Database (NVD)^1^, where they are explicitly verified by developers or security experts. Some studies, including Chakraborty et al. (2021) and Zhou et al. (2019), instead use automated methods to identify VFCs by searching commit messages for predefined keywords related to SVs. Representative datasets employing these two approaches include Big-Vul, which utilizes NVD data, and Devign, which relies on keyword-based filtering.

Jimenez et al. (2018) developed VulData7: an extensible framework and dataset that automatically collects real vulnerabilities from software archives. The current version includes all reported vulnerabilities from the NVD for four security-critical open-source systems: the Linux Kernel, Wireshark, OpenSSL, and SystemD. For each vulnerability, VulData7 provides detailed information, including the vulnerability report (description, CVE number, CWE number, CVSS severity score, and more), instances of vulnerable code (with version lists), and, where available, the corresponding patches (lists of fixing commits) and the code before and after the fix. The framework continuously extracts and integrates data from relevant software archives (using Git and NVD reports) to maintain an up-to-date dataset.

### Security Patch Localization

Once a vulnerability is disclosed, security patch localization focuses on identifying the commit that resolves the vulnerability within a repository. This task is crucial for ensuring that users receive timely updates to protect their systems from potential threats or exploits (Zuo and Rhee 2024). An example of this is PatchScout (Tan et al. 2021), which formulates patch identification as a ranking problem over code commits. To support this, the authors propose using 22 features linking commits and vulnerabilities, categorized into four types: (1) vulnerability identifier, (2) vulnerability location, (3) vulnerability type, and (4) vulnerability descriptive text.

Hermes (Nguyen-Truong et al. 2022) is a tool similar to PatchScout but includes an enhanced method to infer bug tracking IDs even when they are not explicitly mentioned. Despite this improvement, Hermes has only been able to identify 34% of fixing commits in the Project KB dataset, representing a marginal improvement over previous approaches.

Similarly, Wang et al. introduce VCMatch (Wang et al. 2022), a security patch localization method that utilizes 36 statistical features to associate vulnerabilities with patch commits. These features are grouped into four categories: (1) line-of-code (LOC) characteristics, (2) identity attributes, (3) location-based features, and (4) token-based features. Additionally, VCMatch leverages BERT (Devlin et al. 2019) to generate semantic representations of vulnerability descriptions and commit messages. The system trains three classification models–XGBoost, LightGBM, and CNN–and combines their outputs using a voting-based ranking fusion method to enhance prediction accuracy.

Zhou et al. (2021) propose an approach for efficiently managing and identifying security patches, including undisclosed ones, at scale and low cost, using deep neural networks trained on commits from open-source repositories. They compiled security patch datasets that include 38,291 security-related commits and 1,045 CVE patches from four large C-language libraries. Each of the 38,291 commits was manually verified for security relevance. Their system combines two integrated neural networks: a commit-message network that uses pre-trained word embeddings and a code-revision network that learns differences at the statement level. However, the dataset lacks implicit patch samples and includes false negatives due to human labeling errors, which may introduce bias and limit the model’s ability to identify certain types of security patches.

Typically, when a change is identified as security-relevant, an update is quickly released, and users are notified to upgrade to a more secure version of the software. However, some security-relevant changes go unnoticed, representing silent vulnerability fixes. To tackle this, Sawadogo et al. (2022) introduced SSPCATCHER, a co-training-based system for automatically detecting security patches as part of a continuous repository monitoring service. They report that their system can achieve over 80% precision and recall in identifying security patches.

Several research efforts have proposed partial solutions to the patch identification problem. For instance, Tracer (Xu et al. 2022) is a tool that implements an advisory reference crawling technique, analyzing links in security advisories and building a connection graph to detect fixing commits. However, the identified commits are not deeply analyzed, leading to many false positives. Similarly, SAP attempted a related technique using the Project KB dataset but achieved only a 35% success rate (Sabetta et al. 2024).

More recently, FixFinder by Hommersom et al. (2024) introduced a three-phase pipeline to map vulnerability advisories to their fix commits: (1) extract relevant advisory metadata, (2) filter commit candidates using heuristics, and (3) rank them via machine learning (logistic regression). This work is particularly relevant to our study, as Prospector adopts similar input signals but replaces the learned model with expert-defined rules, making the comparison between Prospector and logistic regression especially meaningful.

In parallel, PatchFinder (Li et al. 2024) proposed a two-phase tracing strategy that combined information retrieval and machine learning to locate commits to fix vulnerabilities disclosed in open-source software. PatchFinder first identifies candidate commits using semantic and contextual matching and then refines the ranking with a lightweight classifier. Both FixFinder and PatchFinder confirm the continued interest in accurate, scalable vulnerability-to-patch mapping. However, unlike these fully automated techniques, our work focuses on evaluating the human interpretability and usability of a rule-based tool (Prospector), complementing their algorithmic evaluations with a human-centred perspective.

Vulcurator (Nguyen et al. 2022) applies supervised machine learning to classify commits as vulnerability-fixing or not, using commit-level information such as messages, diffs, and basic file metrics. While it performs better than keyword-based heuristics, it does not capture the broader context or temporal relations that Prospector models explicitly.

More recently, large language models (LLMs) have been introduced to better understand the semantics and intent behind code changes. Yang et al. (2025) integrate multiple sources of context including commit intention, development artifacts (e.g., issue trackers and pull requests), and historical vulnerability data within an LLM-based framework to identify vulnerability-fixing commits which *silently* fix a vulnerability that has not yet being disclosed. This task differs from patch localization as the latter tries to identify a commit in presence of the advisory. Hence the work of Yang et al. (2025) can be seen as an approach towards the identification of security-relevant commits, i.e. *silent* patch localization (Cabrera Lozoya et al. 2021).. Ran et al. (2025) focus instead on security patch localization at industrial scale, fine-tuning LLMs and adopting a sampling strategy similar to Prospector, where the ten commits before and after a confirmed patch are treated as negative examples. These LLM-based methods show strong generalization across projects, but they come with higher computational costs and require careful fine-tuning.

Compared to these approaches, Prospector sits between traditional feature-based techniques and modern LLM-driven systems. It achieves scalability and robustness through structured correlation learning, without relying on extensive natural language input or expensive model adaptation. In this sense, while LLM-based methods extend semantic reasoning, Prospector remains a practical and effective solution for large-scale vulnerability-fixing commit localization.

## Prospector’s Background

To make the paper self-contained, we give a general overview of Prospector and how it works. More accurate details on the tool can be read in the paper by Sabetta et al. (2024).

Prospector is an open source tool whose development was initiated by SAP and further developed in the context of the EU-funded projects AssureMOSS (Grant No.952647) and Sec4AI4Sec (101120393), to which the authors contributed. It has the goal of reducing the effort needed to find security fixes for known vulnerabilities in open-source repositories with a semi-automated approach simplifying and speeding up human review. The tool relies on the idea of crawling relevant resource and information and checking the correlation between the advisory and the commit.

**Fig. 1:**
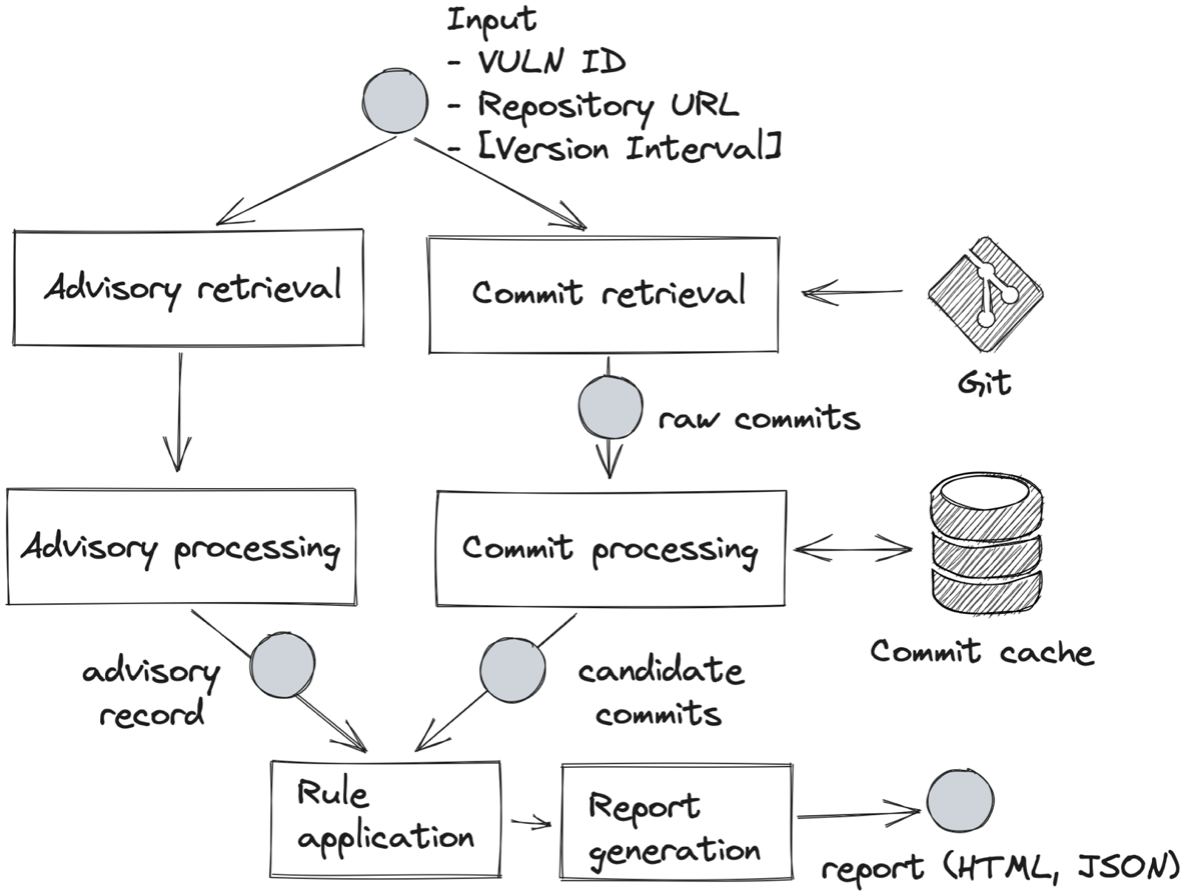
Prospector’s workflow: The figure represents the Prospector’s workflow: (1) advisory retrieval and processing (2) commit retrieval and processing (3) rule application (4) ranking and report generation

Given an advisory expressed in natural language, Prospector analyzes the commits in the target source code repository, ranks them according to a set of predefined rules (by security experts), and generates a report for the user to review in order to identify the commits that resolve the issue. Prospector presents a list of potential candidates in ranked order, with the most relevant commits (those with the highest scores based on matched rules) appearing at the top. The purpose of the experiment is to empirically evaluate whether Prospector effectively assists users in identifying the correct fix commit, particularly by assessing the impact of the ranking order and rules in this identification process (Sabetta et al. 2024). The Prospector workflow can be described in four phases: (1) advisory retrieval and processing, (2) commit retrieval and processing, (3) rule application, (4) and ranking and report generation. The Prospector workflow is shown in Fig. 1 and is described below.

### Advisory Retrieval and Processing

Prospector takes as input the vulnerability identifier and retrieves the advisory from the NVD and processes it to extract relevant information for the subsequent steps. In particular, it gathers the description, timestamps, and references from the advisory. From the description, Prospector uses natural language processing techniques to extract keywords related to the vulnerability, identifies security-related terms by matching against a predefined list, and detects the mentioned file, class, and method names through pattern matching. For references, such as GitHub pages or bug-tracking tickets, it applies parsing techniques to extract any hyperlinks present. These identified keywords, files, classes, methods, and hyperlinks are then used in the rule application phase and play a relevant role in ranking candidate commits.

### Commit Retrieval and Processing

In addition to constructing and analyzing the advisory record, Prospector retrieves a set of candidate commits from the source code repository. The commit retrieval process uses Git to streamline repository cloning and efficiently select subsets of commits. If a version interval is provided, Prospector uses it to retrieve commits with timestamps falling between the specified release dates; otherwise, it selects commits from a time window of 60 days before and after the advisory’s reservation date.

After the candidate retrieval process, each commit is further processed to extract additional information needed for the rule application step (unless these data are already available in the commit cache). This information includes metrics such as the number of hunks, changed files, code modifications, and any references to bug tracking identifiers or GitHub issues within the commit message. Processed commits are stored in the cache.

### Rule Application

Given a processed advisory and a candidate commit, Prospector applies a set of rules to determine whether there is a match, meaning the input meets the criteria defined by the rule. The rules currently implemented in Prospector are summarized in Table 1. For example, the rule CVE_ID_IN_MESSAGE checks whether the vulnerability identifier found in the advisory record appears in the log message of the candidate commit. Similarly, the rule XREF_BUG is satisfied if the commit message of the candidate commit contains a bug-tracking ticket identifier that was extracted during the advisory processing (either mentioned in the advisory or in one of its references).

When a match is found, the candidate commit is annotated with the rule identifier and a plain explanation of the match. These annotations and explanations are included in the final report for the user to review.

**Table 1:** Prospector Rules

Rule Identifier	W.	Rule Description
Cve-Id-In-Message	64	Commits whose message contains only the CVE ID of the vulnerability under analysis. Since this means that there is a direct link between the commit and the vulnerability advisory, this rule is assigned the highest relevance score.
Commit-Mentioned-In-Reference	64	Commits that are mentioned in any of the advisory references. Even if the link is not directly referenced and it should have a lower relevance score, due to the selection process done on the references, this rule is assigned the highest relevance score.
Cross-Referenced-Jira-Link	32	Commits whose message mentions a Jira issue ID referenced by the advisory. Since this means that there is an indirect link between the commit and the vulnerability advisory, this rule is assigned the second-highest relevance score.
Cross-Referenced-Gh-Link	32	Commits whose message mentions a GitHub issue referenced by the advisory. Since this means that there is an indirect link between the commit and the vulnerability advisory and the commit, this rule is assigned the second-highest relevance score.
Cve-Id-In-Linked-Issue	32	Commits whose message mentions a GitHub issue or a Jira issue that contains the CVE ID. Since this means that there is an indirect link between the commit and the vulnerability advisory and the commit, this rule is assigned the second-highest relevance score.
Changes-Relevant-Files	8	Commits that modify files with names containing one or more file names extracted from the advisory text using regular expressions and looking for specific patterns. Since this extraction is more reliable than the keywords extraction, this rule is assigned with a higher relevance value.
Changes-Relevant-Code	8	Commits modifying code that contains one or more advisory relevant words.
Relevant-Words-In-Message	8	Commits whose message contains one or more advisory relevant words.
Adv-Keywords-In-Files	4	Commits that modify files whose names contain one or more advisory keywords. The advisory keywords are extracted from the advisory description and are used to match the lexical context between the advisory and the commit messages. However, this feature is not entirely reliable as it uses basic natural language processing techniques. As a result, the relevance score assigned to this rule is the second lowest.
Adv-Keywords-In-Msg	4	Commits whose message contains one or more keywords extracted from the advisory.
Security-Keywords-In-Msg	4	Commits whose message contains one or more security-related keywords. Some commit messages may provide detailed information about their changes, including security-related details. In such cases, Prospector assigns the second-lowest relevance score to the commit. Currently, 40 security keywords are used for this rule, and they are hardcoded in the *helpers.py* file.
Security-Keyword-In-Linked-Gh-Issue	4	Commits pointing to a GitHub issue or pull request containing one or more security-related keywords. Issues or pull requests often include discussions that may provide additional information about the reasons behind the commit or the problem being addressed.
Security-Keyword-In-Linked-Jira-Issue	4	Commits linked to a Jira issue containing one or more security-related keywords.
References-Gh-Issue	2	Commits whose message mentions a GitHub issue. Developers on GitHub can use issues to report bugs or propose modifications. When a commit message refers to an issue, it usually contains a link using the issue number (e.g., #336). However, those references are common and may not provide much useful information, so the assigned relevance score for this rule is the lowest.
References-Jira-Issue	2	Commits whose message mentions a Jira issue. Developers may use Jira to track issues, bugs, and tasks in the same way they use GitHub issues. When a commit message refers to a Jira issue, it usually contains a unique identifier (e.g., ABC-123). However, referencing a Jira issue in a commit message does not necessarily mean the commit is related to a security fix or a vulnerability. Therefore, this rule is assigned the lowest relevance score.
Commit-Has-Twins	2	Commits that have at least one corresponding *twin* in the repository. Because of the way large repositories are managed, it is common for commits to have twins, even if they are not related to security issues. Therefore this rule is assigned the lowest relevance score.

### Ranking and Report Generation

**Fig. 2:**
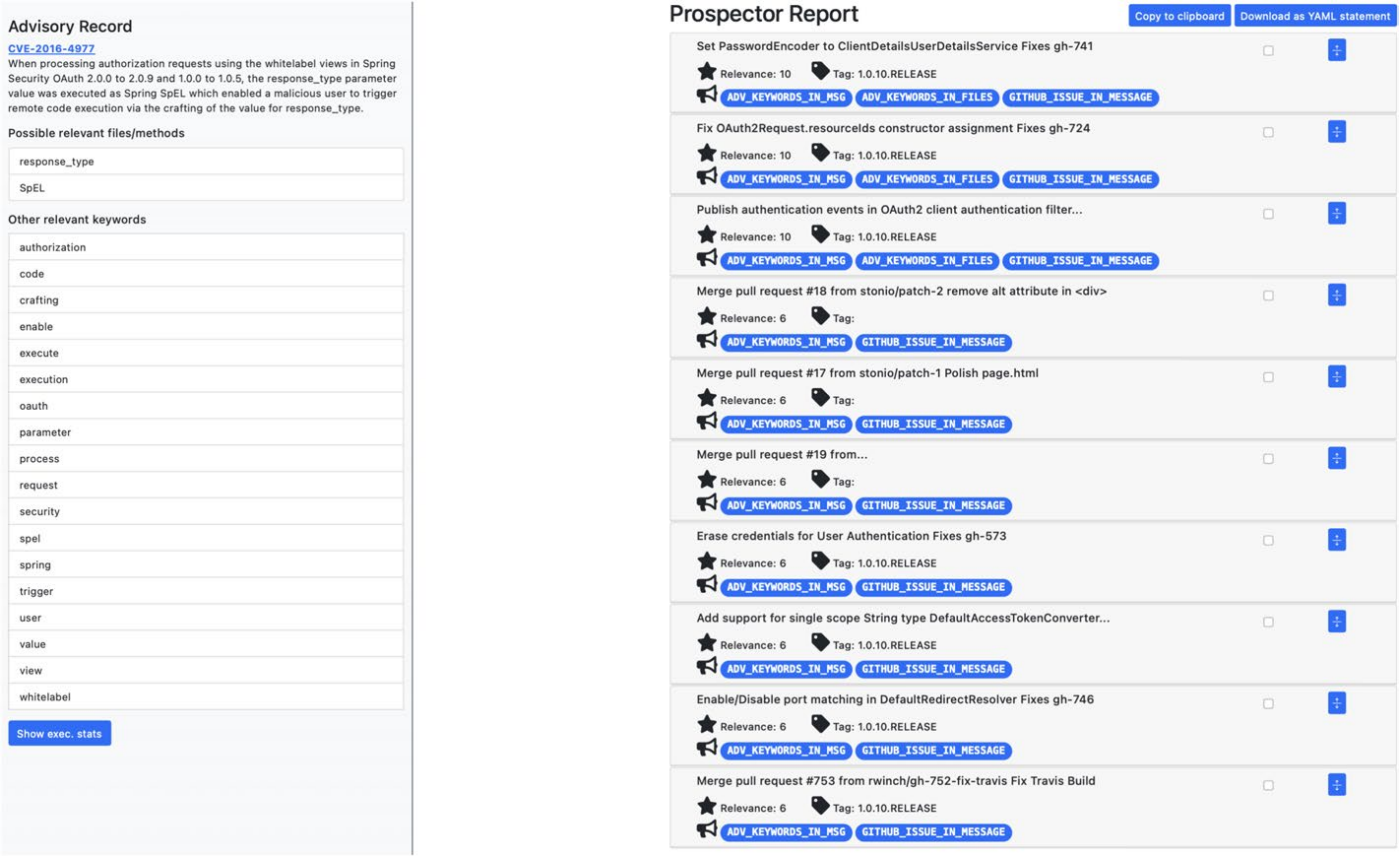
Example of a Prospector report page. On the left at the top of the page there is the CVE ID and the description taken from the NVD page; below there is a list of relevant keywords related to the vulnerability. On the right side of the page there is the list of commits retrieved by Prospector; for each commit is reported the version, the relevance score and the rules matched. It is possible to expand the commit object (with the blue arrow on the side) to read further information as the entire commit message, and the GitHub link of the commit

After the rule application phase, the annotations for each candidate commit are used to rank them. Each rule is assigned a relevance weight (second column (weight) in Table 1) on a logarithmic scale, where a higher weight indicates greater confidence that the commit represents the vulnerability patch. This logarithmic scale helps to distinguish between strong rules (those with $W \ge 32$) and weak rules, ensuring that multiple weak rules do not collectively outweigh a single strong rule in the ranking process. The current weight values are based on the expert knowledge. To rank the candidates, a relevance score is calculated by adding the weights of all matched rules. The results are compiled into a report that lists the candidate commits in descending order of relevance, thereby prioritizing the commits most likely to address the identified vulnerability. Figure 2 shows an example of a report produced by Prospector.

## Research Methodology

In this section, we introduce our research questions and detail our experimental design.

### Research Questions

We structure our study around three research questions. We aim to understand whether (1) expert-defined rules have performance comparable to traditional ML models, (2) expert-defined features reported by the tool are consistent with actual usage, and finally (3) investigate the performance of junior assessors when using the tool.

#### Analysis of Expert-Defined Common Sense Rules to ML Models

We first perform a study to control the performance of expert-defined rules with traditional ML models, to make sure the human participants do not fail just because the tool does not work; therefore, we define our first research question as follows:
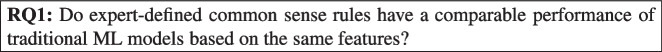


To answer RQ1 we ran four different machine learning models: linear regression, logistic regression, XGBoost, LightGBM. We decided to use the first two as they are the most common ML models, and we selected XGBoost and LightGBM as they are two of the three models used by VCMatch. To perform all these models we used the library provided in the python environment and we trained and tested them on ProjectKB dataset; the model were trained to predict the vulnerability patch. Although deep learning (DL) approaches are widely used for commit classification tasks, we deliberately exclude them from this study due to the limited size of the available dataset. The ProjectKB dataset comprises 1,282 fixing commits associated with 624 vulnerabilities across 205 projects, which is insufficient for training a DL model without severe overfitting. According to the empirical scaling laws reported by OpenAI (Kaplan et al. 2020), the number of training examples required grows proportionally with the number of model parameters. Even for a relatively small model with around $10^2$ parameters, at least 150,000 training samples would be needed to achieve generalization. Our dataset is two orders of magnitude smaller, making any DL-based evaluation unreliable. Furthermore, alternative datasets commonly used in the literature (e.g., CVEfixes, MoreFixes, PatchDB) are unsuitable, as they are constructed through explicit CVE–commit references that would make Prospector trivially correct by design. For these reasons, we focus instead on interpretable machine learning models and human-centered evaluation, which better align with the objectives of this study.

We did not run any deep learning model due to the size of the training dataset, and we did not use any large language model (LLM) because the dataset used for the training is dated before the LLM models; therefore, there is a data leakage as the latest have already been trained on Prospector.

To compare Prospector’s performance respect to the chosen ML models, for each technique we calculate precision, recall, accuracy and chance accuracy (Doswell et al. 1990) or Heidke skill score (HSS). The chance corrected accuracy is the value of accuracy in which we have eliminated the number of elements we would have identified purely due to chance which is way higher than 50% if the data is not balanced. We define the last one as follows (Hyvärinen 2014):

1 $$\begin{aligned} chance\_positive = (TP + FP) \times \frac{(TP + FN)}{TN + TP + FP + FN} \end{aligned}$$

2 $$\begin{aligned} chance\_negative = (TN + FN) \times \frac{(TN + FP)}{TN + TP + FP + FN} \end{aligned}$$

3 $$\begin{aligned} found\_by\_chance = chance\_postive + chance\_negative \end{aligned}$$

4 $$\begin{aligned} HSS = \frac{(TN + TP - found\_by\_chance)}{(TN + TP + FP + FN) - found\_by\_chance} \end{aligned}$$

Chance accuracy is particularly relevant as it has been proposed by weather forecasts experts for addressing the bias of traditional metrics in presence of very rare events (Doswell et al. 1990).

We then used the Python SHAP library 2 to compare the weights of each rule given by the company security expert to the one obtained from the ML analysis.

To normalize the values, we divide all the Shapley values for each regression type by the difference between the highest and lowest Shapley values and multiply by the highest score of the expert defined rules, which is 64. Values are rounded to the nearest integer. We calculate the normalization as follows:

5 $$\begin{aligned} Weight_i = \left[ \frac{SHAP_{i}-SHAP_{min}}{SHAP_{max}-SHAP_{min}}\right] *(Prospector_{max}-Prospector_{min})+2 \end{aligned}$$

Where the $Prospector_{max}$ is the highest score of the expert defined rules, and $Prospector_{min}$ is the lowest, and they correspond respectively to 64 and 2.

For example, the normalized Shapley value of COMMIT_MENTIONED_IN_REF-ERENCE for linear regression of 0.71 is the highest value obtained and is therefore assigned the weight 64. The CVE_ID_IN_MESSAGE has a Shapley value of 0.5 and its weight equals 45 = 0.5/0.71*64.

To make rules comparable, we put side by side the weight of the expert-defined rules and the Shapley values obtained by predicting a fixing commit for each ML model.

Once the tool has selected the first top ten most likely security fixing commits (possibly even failing to have a fixing commit, or having multiple ones), how humans decide or act to select the fixing commit? In this respect, we continue the rest of our research by analyzing two different users: senior security experts and junior assessors (i.e., MSc students).

#### Analysis of Expert Assessment

One of the main goal of our study is to understand whether the expert-defined rules are consistent with usage. We formulate our second research question as follows:
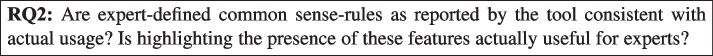


To answer RQ2, we performed seven think-aloud interviews with senior employees from software companies. We recorded the interviews and then wrote the full transcript. To analyze the interviews, we adopted the *applied thematic analysis* (Guest 2012), following the principle of emergence (Gregory et al. 2015), according to which the data gain relevance in the analysis through systematic generation and iterative conceptualization of codes and concepts. We also identified a common pattern and tasks computed by the seven participants to define “the process” to identify the vulnerability patch.

#### Experiment with Junior Assessement

Our last main goal is to understand whether the rules and the order of the vulnerability patches identified by Prospector are informative for junior assessors (such as MSc students). We defined our third research question as follows:
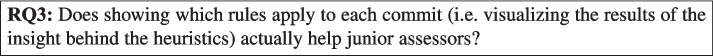


To answer RQ3, we performed the Chi-square test to determine which Prospector’s feature is statistically significant to identify the fixing commit. Finally, to consolidate the answer for RQ3, we proposed to a small group of junior participants to analyze the same eight reports shown to the senior participant. They had five minutes for each report to perform the task.

This experiment scenario is the same as proposed in the think-aloud interview as we wanted to replicate a “real-world scenario” and analyze how users with less experience perform differently when using Prospector. We have used the Agresti-Coull-Wilson rule for confidence interval (Agresti and Coull 1998) to estimate the confidence interval 95% for the proportion of correct fixing commit identified 3

We obtained the upper and lower bounds of the confidence interval by solving *p* from (6), where $\hat{p}$ is the sampled proportion of the correct patch in the manually validated sample, $z_{a/2} = 1.96$ for the two-sided confidence interval 95% and *n* is the sample size, i.e., the total number of Tested Patches in the sample.

6 $$\begin{aligned} |\hat{p}-p| = z_{\alpha /2}\sqrt{\frac{p(1-p)}{n}} \end{aligned}$$

### Experimental Design

#### Think Aloud Execution Plan

The participants were interviewed through the Zoom platform and the sessions lasted forty to eighty minutes. The first phase of the interview focused on asking questions about the background of the participants and their experience using Prospector as users. In particular, we asked three questions: (1) *“How much experience do you have with security vulnerabilities and for how long have you been doing security vulnerability detection?”*; (2) *“For how long have you been working with Prospector output? Like using it as a user? ”*; (3) *“What is the procedure that you follow to identify the security fixing commit?”*.

In the second phase of the interview, we shared with them a Qualtrics survey with eight different Prospector reports showing the CVEs that we selected (§ 4.5). The participants had 5 minutes to answer all the questions for each report, but we gave them additional time to respond when needed to collect all their observations. Each report contained the first ten commits extracted by Prospector, we preserved the ranking order and all the information collected by the tool, as we wanted to perform the experiment replicating a “real-world” scenario.

#### Perception Analysis

In the last phase of the experiment, we collected some feedback about the overall experience to better define the experiment design with the junior assessors. Therefore, we asked whether (1) the shown reports are useful for Prospector’s purpose, (2) the task to perform was clear enough, and (3) the given time was enough to identify the commit.

#### Junior Assessment Execution Plan

The first step of the experiment consist of a *training* phase. In previous studies in which professionals were involved, the minimum duration of the training was 1.5 hours (Allodi et al. 2020; Gramatica et al. 2015). This is also essentially a typical session of a professional training session performed in industry^4^. Since we used participants with less expertise, we considered a longer training duration based on the finding from the survey by (Kollanus and Koskinen 2009). The authors mention the use of “overview meetings” in most of the software inspection publications, which seems to imply that the duration was significant (as a presentation of 10-30 minutes would hardly be considered a “meeting”).

All participants completed two training phases (of around 3 hours) on finding vulnerabilities in the source code. These training sessions prepared the participants for studies that focused on the identification of vulnerabilities in a source code. In particular, the first training session focused on four specific vulnerabilities types selected by OWASP top 10 2017 (Path Traversal, User Injection, XSS, DoS). Therefore, we selected CVEs characterized by one of these types.

The participants completed a 45 minute training phase about the Prospector tool Prospector. The session focused on showing the research problem that the company addressed, what is the purpose of the tool and how it is a possible solution to the research problem, and general information about how Prospector works. In the last part of the training phase, the participants performed an example exercise to gain confidence with the experiment process and familiar with what to expect. Digital copies of lecture slides, technical documentation, etc. were provided to the participants, and they could also be consulted at any time during the experiment.

The *execution* part of the experiment consists of two phases. The first phase of the experiment lasted an hour of physical laboratory, the participants had 10 minutes to complete each task. Each room was supervised by an experimenter whose role did not go beyond the supervision of the room and the technical support, s/he could not reply to questions regarding the solution.

The experiment was carried out with the support of Qualtrics to collect the responses of the participants. We created a survey that showed to the participants six different Prospector reports, each report being about one of the CVEs selected (§4.5). In that report, the top 10 commits filtered by the tool were shown, and for each report the participants were asked to identify the fixing commit for the CVE in question. We also asked whether the order of the commits, or the rules (if present) were informative in identifying the answer.

We designed a balanced orthogonal design which is also known as Taguchi design (Massacci et al. 2024) where each participant is randomly assigned to one out of six groups as shown in Table 2 where each participant goes through the evaluation of reports in which they have to analyze 6 different *scenarios* (i.e Prospector reports). The demonstration of the balancing of our particular design is available on our artefact repository.

**Table 2:** Experiment design. X*n*: X is the Prospector report type (C: control group/ commits in temporal order and no rules, R: commits in relevance order but no rules (keywords), K: commits in temporal order and with matched rules (keywords)), *n* is the Prospector report ID (one for each CVE)

	Consecutive Sessions					
Groups	S.I	S.II	S.III	S.IV	S.V	S.VI
Group A	C1	R3	K5	K2	R4	C6
Group B	K4	C2	R1	R6	C5	K3
Group C	R2	K6	C4	C3	K1	R5
Group D	R5	K1	C3	C4	K6	R2
Group E	K3	C5	R6	R1	C2	K4
Group F	C6	R4	K2	K5	R3	C1

For example a participant assigned to group A will analyze

1. controls with commits in temporal order and no rules (C) on the scenarios corresponding to CVEs 1 and 6
2. commits in relevance order but no matched rules (R) on the scenarios corresponding to CVEs 2 and 5
3. commits in temporal order and matched rules (K) on the scenario corresponding to CVEs 3 and 6

This design ensures that the experiment is fully balanced: everybody sees the same numbers of controls, relevance, and rules report type and each group is exposed to an equal number of orderings across all group: e.g the number of times a control is shown before a relevance order report and the number of times a report with matched rules is shown before a control are equal.

For the second phase of the experiment that we refer as *validation* phase, we showed to the participants eight additional Prospector reports, which are the same shown in the think-aloud interviews to the senior assessors. In this phase they had only 5 minutes to inspect each report and we did not create any treatments. The idea was to use the tool as is and create a real-world scenario.

#### Perception Analysis

We collected *feedback* about the experiment, therefore the participants were asked to respond questions about their perception of the task. The questions were answered through an ordinal scale; moreover, we also design an open question for those who want to give additional comments, and give suggestions.

### Experimental Variables

Table 3 shows the independent and dependent variables that we collected for the think aloud interviews, and the juniors assessment.

During the first phase of the think-aloud interview, we collected the background variables *Knowledge of Security Vulnerabilties* and *Knowledge of Prospector*. The first describes the experience of the participants in detecting/fixing security vulnerabilities, while the second describes their experience in using Prospector as users.

**Table 3:** Experimental Variables

Name	Description	Operationalize
*Independent variables (design)*		
Assignment to a Treatment	Random assignments of the participants to a treatment (control, no rules, no relevance).	Nominal (A)
*Background variables*		
Knowledge of Java	Self-reported concrete experience on Java	Ordinal scale (B)
Knowledge of Security Vulnerabilities	Self-reported concrete experience on vulnerability assessment	Open question
Knowledge of Prospector	Self-reported concrete experience on the usage of Prospector	Open question
*Dependent variables*		
Fixing Commits Found	Numbers of correct fixing commits identified by the participants	Multiple Choice (C)

**(A)** Automatically performed by the Qualtrics submission tool; **(B)** Multiple choice: no experience, slightly familiar, somewhat familiar, really familiar, expert; **(C)** selection choice of the correct fixing commit

In the experiment performed with the master students we collected the independent variable *Assignment to a Treatment* in the first phase of the experiment, which describes the random assignment of a participant to one of the three treatments: (1) control, (2) no rules and relevance order, (3) rules and temporal order. We also collected the main dependent variables *Fixing Commits Found* to measure the number of correct fixing commits identified and answer RQ3.

### Ethical Approval

For the experiment with MSc students, the ethical procedure was followed at Institution XXX and it was determined that a full ethical review was not necessary. In particular, this was determined because (i) upfront opt-in consent was asked, (ii) no personal or sensitive information was involved, (iii) it did not pose potential risks to either participants or researchers, (iv) the confidentiality of the participants was guaranteed by collecting data by GDPR compliant tool and removing their details before processing the data for the analysis, (v) the incentives to participate were minimal (see below), (vi) and the participants were not deceived and thoroughly debriefed afterwards (they actually had full access to the anonymized data). There was no monetary compensation, and the participants received a compensation in terms of coursework bonus. This value was minimal (less than 2% of the final grade), and the participants could deny consent and still receive the participation bonus. Personal details (name and email) were only collected to grant the coursework’s bonus.

The choice of the Taguchi design is also a good design from an ethical perspective (along the benefice and justice dimension) considering the use of students as particpants. In this way, every student has the possibility of having a learning experience using a professional tool in a concrete scenario.

### Experimental Objects

#### Choice of the Dataset

We selected ProjectKB dataset as it aligns with the primary goal of our study, which is to evaluate the performance of Prospector’s rule-based approach under controlled and manually curated conditions. The dataset provides verified mappings between vulnerabilities and their corresponding fix commits, allowing for a realistic assessment of precision and recall. Infact, other available datasets such as *CVEfixes*, *MoreFixes*, *PatchDB*, are constructed by linking CVE identifiers directly to GitHub commits through explicit references in advisories. In such cases, Prospector’s highest-weight rule (CVE_ID_IN_MESSAGE or COMMIT_MENTIONED_IN_REFERENCE) would always be satisfied. As a consequence, the tool would achieve 100% precision and recall by design, leading to a biased and non-informative evaluation.

For instance, in datasets like *CVEfixes*, every entry is derived from CVE advisories that already contain a GitHub commit URL pointing to the fix. Under these conditions, Prospector would trivially identify all commits as correct, since its strongest heuristic rule exactly matches the construction logic of the dataset. Therefore, such datasets cannot be used to provide a meaningful comparison, as they would not capture realistic false positives or false negatives. We also found a recently introduced dataset, MoreFixes (Akhoundali et al. 2024), where the vulnerability patches were actually identified using Prospector itself. This means the dataset is essentially built on Prospector’s decisions, making it unusable for our evaluation because it would introduce circular reasoning and completely bias the results.

ProjectKB overcomes this limitation. Its instances do not always include explicit GitHub URLs or CVE identifiers in commit messages, allowing a fair assessment of Prospector’s heuristics and ranking strategy. This choice ensures that the evaluation reflects real-world conditions in which commits may lack direct references or clear textual cues.

Although the data set is smaller than those used in deep learning studies, this trade-off is consistent with our research goals. Our objective is to assess the viability and interpretability of rule-based heuristics rather than to train large models that require extensive data. In future work, we plan to extend this evaluation to larger datasets–after filtering out trivial cases where CVE identifiers or commit URLs are explicitly provided–to further analyze Prospector’s scalability and its potential integration with learning-based techniques.

#### ProjectKB

ProjectKB is a vulnerability dataset that contains a manual collection of plain text files that provide details about each vulnerability, including references to fixing commits. The data were obtained both from the National Vulnerability Database (NVD) and from project-specific web resources. The data set consists of 624 publicly disclosed vulnerabilities that affect 205 different open-source Java projects, on the 1282 commits that fix them 5 (Ponta et al. 2019).

#### CVEs for the Expert Evaluation

We selected eight different CVEs from the ProjectKB dataset. For the selection process, we implemented a Python script to select all CVEs whose ground truth according to the Tracer data set (Xu et al. 2022) has only one fixing commit that appears in the first 10 commits of the list. To narrow down the selection, we computed the Spearman rank correlation coefficient between the different rules to identify which rules have the highest correlation for the fixing commits. We define the rule with highest correlation as “top predictor”, which in our case is the rule COMMIT_IN_REFERENCE. When this rule is matched, it indicates a higher likelyhood that the commit is the one fixing the vulnerability. The second predictor are the rules with the second highest correlation, which are the rules CVE_ID_IN_MESSAGE and CROSS_REFERENCED_GH_LINK. Finally, we define the third predictor the rules RELEVANT_WORDS_IN_MESSAGE and SEC_KEYWORDS_IN_MESSAGE. Given these definitions, we state the following criteria to select the CVEs:

1. The top predictor is wrong, therefore the rule is matched for a specific commit but it not the one fixing the vulnerability
2. The top predictor is missing, but the commit is fixing the vulnerability. Also, the second and third top predictors of the list are matched for that specific commit.
3. The top predictor is missing but the commit is fixing the vulnerability. The second top predictor is matched, but the third top predictors is not.
4. The top predictor is matched and the commit is fixing the vulnerability, but the commit is not in the first position of the list.

In Table 4 we report the CVEs selected for the evaluation of security experts.

**Table 4:** CVEs selected for the Experts Evaluation

	Criteria
CVE-2013-1880	3
CVE-2015-2912	4
CVE-2016-4977	2
CVE-2017-15703	3
CVE-2018-14574	4
CVE-2018-1000854	2
CVE-2018-1000872	3
CVE-2019-6975	1

#### CVEs for Junior Assessment

Table 5 shows the six different CVEs that we selected for the experiment. The CVEs have been selected by a security expert from SAP according to three requirements: (1) two CVEs whose fix commit is in the first three positions in the report produced by Prospector, (2) two CVEs whose fix commit is in the positions from 4 to 10, (3) and two CVEs whose fix commit is not present in the commits list from the report. All CVEs come from the ProjectKB dataset. The selection criteria for the assessment with junior assessment are different from those with experts because in this phase of the study our goal was to evaluate whether Prospector is useful to the user in the “last mile” step. To do this, we selected criteria needed to focus on evaluating the tool features such as the rules, and the ranking order.

**Table 5:** CVEs selected for the Junior Evaluations

	Fix Commit	Position
CVE-2019-17567	Backport to v2.4: *) mod_proxy_http: handle upgrade/tunneling...	3
CVE-2019-0207	In resource code, handling backslash as path separator better	3
CVE-2019-12407	2.11.0-M5-git-02 [JSPWIKI-1111] WYSIWYG xss fixes, web app manifest	5
CVE-2019-3810	MDL-64372 userpix: Escape fullname string on userpix index page	10
CVE-2020-1937	fixed commit not in the first 10	n.a.
CVE-2019-0219	fixed commit not in the first 10	n.a.

For the later validation phase we also use the same vulnerabilities used by the senior experts.

## RQ1: Performance Comparison of Common Sense Rules Defined by Experts with Traditional ML Models

### Results

In Table 6 we report the results of the performance of expert-defined rules (the method adopted by Prospector), and of the four ML models that we selected: (1) linear regression, (2) logistic regression, (3) XGBoost, and (5) LightGBM. For all the techniques we did ten rounds of training with 80/20 splitting method and testing; as final result we calculated the average precision, recall, accuracy and HSS of all rounds.

**Table 6:** Comparison of performance of the expert-defined rules, linear regression, logistic regression, XGBoost, and LightGBM

Method	Precision	Recall	Accuracy	Accuracy*
Expert-defined rules	0.69	0.94	0.99	0.79
Logistic regression	0.80	0.78	0.99	0.81
Linear regression	0.77	0.83	0.99	0.79
XGBoost	0.69	0.94	0.99	0.82
LightGBM	0.83	0.83	0.92	0.80

**Fig. 3:**
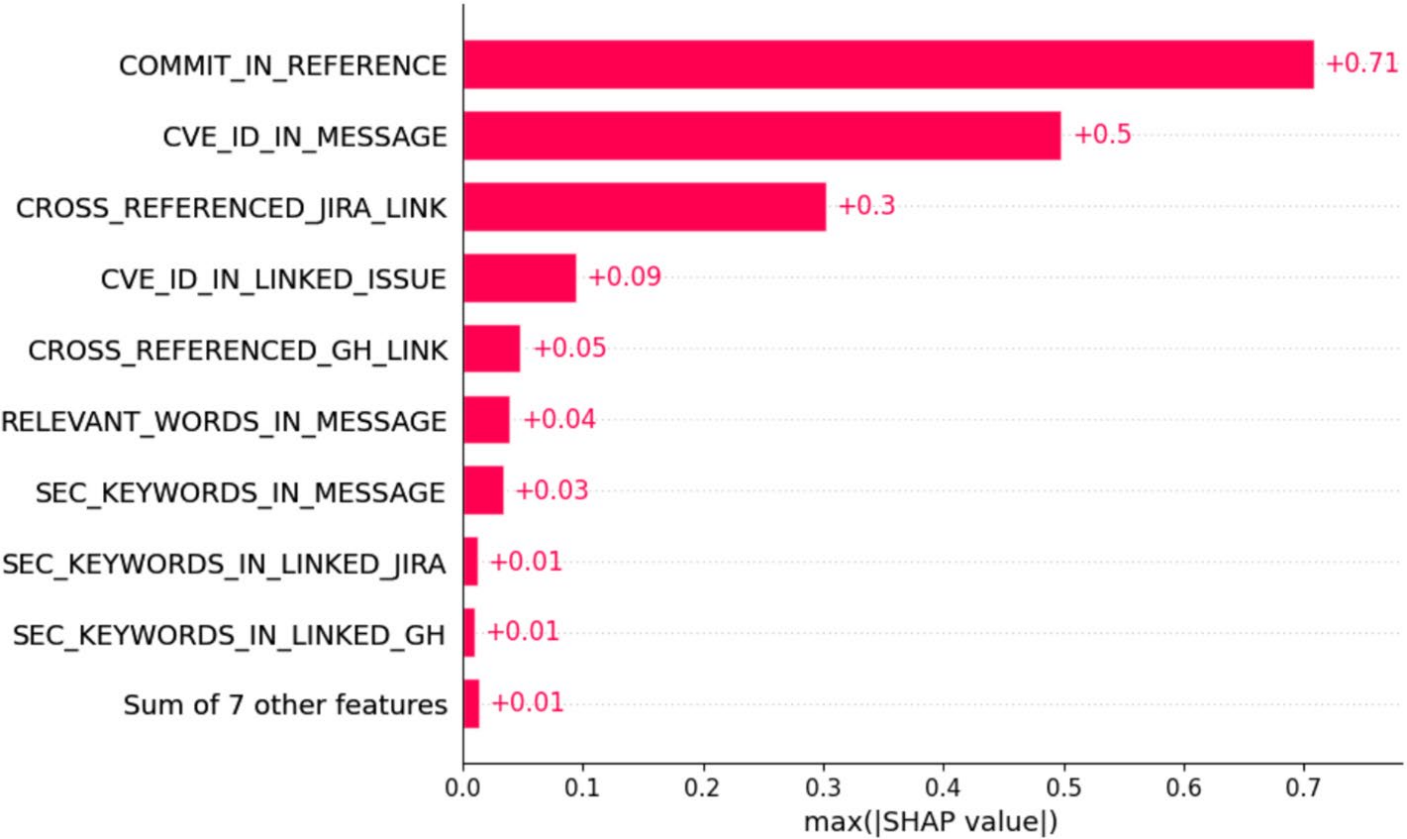
Shapley Values for Linear regression

Subsequently, we used the SHAP library to compare the weights defined by the security experts associated to the Prospector rules, with the Shapley values obtained for each ML model from the analysis. Figure 3 shows an example of the Shapley values for the linear regression, we then normalized the values as explained in Section 4.

Table 7 shows the results we obtained, from it we can observe that the weights defined by the experts for the rules with highest score are similar to the values obtained through the ML analysis. Therefore, all models agree on which rules are the most relevant (COMMIT_MENTIONED_IN_REFERENCE and CVE_ID_IN_MESSAGE), but for the other rules we observe an optimistic evaluation compared to the expert weights with logistic regression, and XGBoost, which can be a problem, as it might lead to overfitting issues.

**Table 7:** Prospector Rules vs ML

Rule Identifier	Expert	Lin.	Log.	XGB	LGBM
CommitMentionedInReference	64	64	64	64	61
CveIdInMessage,	64	45	56	63	64
CrossReferencedGhLink	32	33 $\uparrow$	31	55 $\uparrow$	35 $\uparrow$
CrossReferencedJiraLink	32	13 $\downarrow$	41 $\uparrow$	64 $\uparrow$	36 $\uparrow$
CveIdLinkedIssue	32	2 $\downarrow$	2 $\downarrow$	46 $\uparrow$	9 $\downarrow$
ChangesRelevantFiles	8	10 $\uparrow$	34 $\uparrow$	11 $\uparrow$	15 $\uparrow$
ChangesRelevantCode	8	10 $\uparrow$	33 $\uparrow$	2 $\downarrow$	23 $\uparrow$
RelevantWordsInMessage	8	12 $\uparrow$	40 $\uparrow$	45 $\uparrow$	18 $\uparrow$
AdvKeywordsInFiles	4	9 $\uparrow$	31 $\uparrow$	39 $\uparrow$	2 $\downarrow$
AdvKeywordsInMsg	4	10 $\uparrow$	36 $\uparrow$	39 $\uparrow$	17 $\uparrow$
SecurityKeywordsInMsg	4	12 $\uparrow$	41 $\uparrow$	39 $\uparrow$	20 $\uparrow$
SecurityKeywordInLinkedGhIssue	4	9 $\uparrow$	22 $\uparrow$	40 $\uparrow$	19 $\uparrow$
SecurityKeywordInLinkedJiraIssue	4	10 $\uparrow$	31 $\uparrow$	34 $\uparrow$	14 $\uparrow$
ReferencesGhIssue	2	9 $\uparrow$	6 $\uparrow$	13 $\uparrow$	4 $\uparrow$
ReferencesJiraIssue	2	9 $\uparrow$	15 $\uparrow$	11 $\uparrow$	10 $\uparrow$
CommitHasTwins	2	9 $\uparrow$	31 $\uparrow$	43 $\uparrow$	5 $\uparrow$

In the table below we provide the original weight of the rules (Expert column) and the transformed weight according to the SHAP values of linear (Reg. column). We indicate with $\uparrow$ and $\downarrow$ the value when its use would generate an inversion in the order between the ML algorithm and the common sense algorithm. Moreover for each rule we report the SHAP values min, max and mean

For some other rules with lower scores there is a slight difference in the ranking proposed by the ML models but without making significant changes, expect for the logistic regression, and XGBoost model as they tent to increase the scores also of rules with lower scores according to the security experts (e.g. ADV_KEYWORDS_IN_FILES, ADV_KEYWORDS_IN_MSG, etc.). This might be a problem as it could lead to an overfitting issue. In Table 8 we show the changes in order of position of the ML models, compared to the list proposed by the experts.

**Table 8:** Expert defined rules ranking vs ML defined rules ranking

Rule Identifier	Expert	Lin.	Log.	XGB	LGBM
CommitMentionedInReference	64	0	0	0	0
CveIdInMessage,	64	0	0	0	0
CrossReferencedGhLink	32	0	0	1	0
CrossReferencedJiraLink	32	-1	0	1	0
CveIdLinkedIssue	32	-4	-4	1	-2
ChangesRelevantFiles	8	0	2	0	1
ChangesRelevantCode	8	0	2	-2	2
RelevantWordsInMessage	8	1	2	2	1
AdvKeywordsInFiles	4	1	3	3	-1
AdvKeywordsInMsg	4	1	3	3	2
SecurityKeywordsInMsg	4	2	3	3	2
SecurityKeywordInLinkedGhIssue	4	1	2	3	2
SecurityKeywordInLinkedJiraIssue	4	1	3	3	2
ReferencesGhIssue	2	2	2	3	1
ReferencesJiraIssue	2	2	3	2	2
CommitHasTwins	2	2	4	4	1

#### Takeaway

The rules are consistent, and the explainable features are essentially the same. There appear to be no additional features identified by the machine learning models that were not already captured by the experts. Therefore, another state-of-the-art tool would likely select a similar set of vulnerabilities, probably for the same underlying reasons, to present to the human analyst.

Since the tool performs consistently and does not introduce failures in its responses, we can confidently move on to the next part of the study: the human evaluation phase.

## RQ2: Rules Consistency with Usage

### Demographics and Expertise - Senior Participants

In the first part of the interviews, we asked some questions about the background of the participants to validate the expertise and the potential impact that the latter may have on the outcome of the experiment.

In particular, we first asked: * “How much experience do you have with security vulnerabilities and for how long have you been doing security vulnerability detection?”*. Participant 1 said between 8-10 years, participant 2 stated: *“I have been working on that for almost 10 years, and in particular, regarding vulnerability detection, (...) four years. Between 2015 and 2019.”*. Participants 3, 4, and 5 said they have worked in security-related fields between four and five years. Participant 6 stated: *“I did all my master’s studies in two years for the master’s studies in Cyber Security at the university.. But then in my current job I don’t really work with vulnerabilities. My only experience comes from university. ”*. Finally, participant 7 said: *“[...] part of my job is assessing whether the vulnerabilities reported in HackerOne by external researchers are actually vulnerabilities or not. [...] and I have been doing that for about a year and a half”*.

Then we asked them how long they have been using Prospector. Participant 1 stated: *“[...] Time-wise, it has been maybe seven years, six years.”*, participant 2 instead said that they never used the tool as a user but were involved in providing feedback for the development of the tool and having a look at the features shown in the reports to check if they match the same information that they would normally identify the vulnerability patch manually. All the other participants they have never used to the tool.

### Results

**Table 9:**
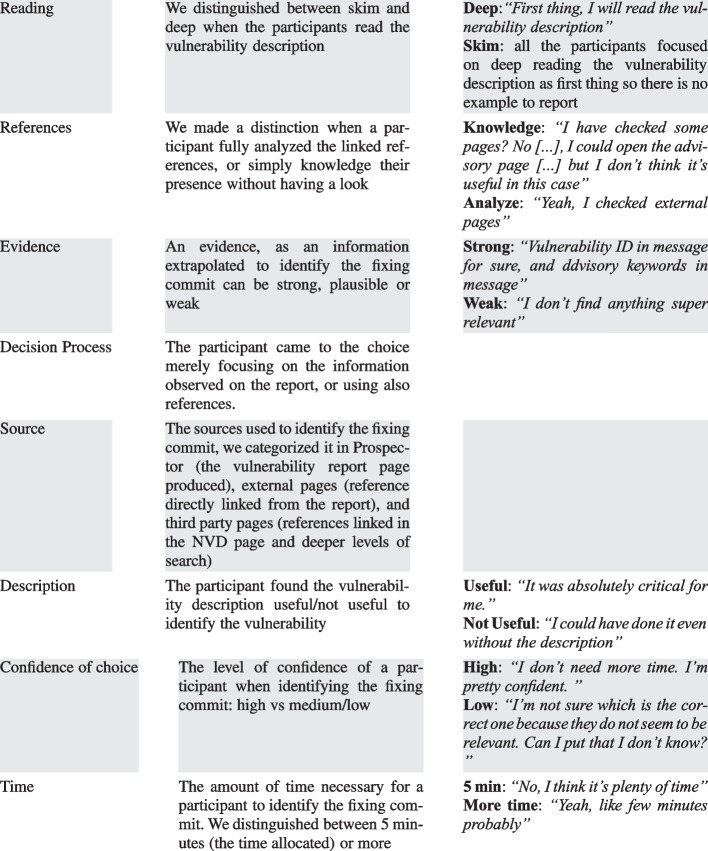
Codes used in the study

To analyze think-aloud interviews, we have adopted the *applied thematic analysis* (Guest 2012), following the principle of emergence (Gregory et al. 2015), according to which the data gain relevance in the analysis through systematic generation and iterative conceptualization of codes and concepts.

#### Codebook Development

From the interviews, we analyzed the observations and behaviors of the participants while inspecting the proposed Prospector reports and classified them according to the categories (codes) that we defined. We consolidated the codes into a coding book that is reported in Table 9. It lists the codes identified in our study and also shows some examples.

The code *Reading* describes the participant’s effort in reading the vulnerability description shown in the report; we made a distinction between skim and deep reading. *References* refers whether the participants simply acknowledge the existence of the references reported in the report but did not analyzed them, or inspected them to gather more information to choose the fixing commit.

With the term *Evidence* we mean the rules/information matched by a commit (e.g. CVE ID in message, GitHub link, etc.), this code describes whether the contribution of an evidence was strong, plausible, or weak in leading the participant to their choice.

The code *Decision Process* indicates the variables that contributed to the decision of the participants. We distinguished the process in (1) reference-based and (2) evidence-based; therefore, we classify whether a participant made their decision relying also on the information gathered through the linked references, which coincides with a deeper analysis of the tool, or simply relied on the evidence/information observed to lead the participant to a faster choice without spending too much time in analyzing the report and the references.

*Source* is classified in: (1) Prospector report, (2) external pages, and (3) third party pages. This code describes the sources used by the participant in the decision process. The distinction between external pages and third-party pages lies in the depth level of references, the firsts refer to the references directly linked in the report (1st level), and the seconds are references of the 2nd level of search (e.g., links in the NVD page).

The last three codes *Description*, *Confidence of choice*, and *Time*, respectively, describe whether (1) the participant found the vulnerability description useful as information, (2) how confident the participant is about their choice (high confidence vs medium/low confidence), and (3) whether the provided time was enough to complete the task or more time was needed to make a decision.

#### Findings

We used scipy.stats.spearmanr^6^ python function to calculate the correlation of the defined codes, we report the obtained results in Table 10.

For some of the codes, there was a perfect inverted correlation, for example, when there is 100% of *references-acknowledge* there is 0% of *references-analyze*. Similarly, when there is 100% *time-5 minutes* there is 0% *time - few more* minutes. These are expected results when two variables are in contrast. We also did not report the results for *evidence-plausible*, and *source-third parties pages* as we did not observe any significant correlation.

We observed that in the presence of strong evidence, the participant did not need more time to make a choice as there is a correlation of -58% between *evidence-strong* and *time - few more* minutes; it is a rare case. Instead, when there is a weak evidence in 51% of the cases, more time is needed from the participant for the final decision.

In presence of a strong evidence we cannot make any assumption whether a description is either useful or useless as there is not a correlation with an explicit judgment. However, in the presence of weak evidence, there is a significant correlation with a useless description.

Regarding the decision process, neither the references nor the evidence have a correlation with the levels of confidence about the choice taken (high, medium, low. This is an interesting observation as we would expect some kind of correlation, for example, we hypothesized that a decision process based on evidence only leads to a high confidence of choice. However, we did not have enough data point to support this hypothesis; therefore, more rounds of interviews would be necessary.

Finally, when a decision process is only evidence based, the participants did not need to look at external pages; moreover, as there is a -57% correlation, it is a rare case that when a decision process is evidence based, the participant needed to look at external pages.

**Table 10:** Correlation of codes across responses

	References	Evidence		Decision Process		Source	Description		Confidence			Time
	A.	S.	W.	R.	E.	E.P.	U.	N.U.	H.	M.	L.	F.M.
A.	100%*	-16%	10%*	79%*	-68%*	93%*	14.%	15%	-28.%	21%	14.%	22%
S.	-16%	100%*	-83%*	-8%	23%	-13%	-6%	-34%	71%*	-41%*	-52%*	-58%
W.	10%	-83%*	100%*	13%	-19%	8%	-5%	43%*	-62%*	33%	49%*	51%
R.	79%*	-8%	13%	100%*	-79%*	72%*	10%	17%	-9%	-1%	15%	15%
E.	-68%*	23%	-19%	-79%*	100%*	-57%*	-12%	-13%	11%	-4%	-12%	-16%
E.P.	93%*	-13%	8%	72%*	-57%*	100%*	19%	14.%	-26%	20%	13%	19%
U.	14.%	-6%	-5%	10%	-12%	19%	100%*	-53%*	-1%	10%	-12%	4%
N.U.	15%	-34%	43%*	17%	-13%	14.%	-53%*	100%*	-32%	7%	39%*	36%
H.	-28.%	71%*	-62%*	-9%	11%	-26%	-1%	-32%	100%*	-76%*	-50%*	-78%
M.	21%	-41%*	33%	-1%	-4%	20%	10%	7%	-76%*	100%*	-18%	56%
L.	14.%	-52%*	49%*	15%	-12%	13%	-12%	39%*	-50%*	-18%	100%*	43%
F.M.	22%	-58%*	51%*	15%	-16%	19%	4%	36%	-78%*	56%*	43%*	100%

The table shows the correlation of codes among the population, each row and column represent a rule: References - Analyze (A.), Evidence - Strong (S.), Evidence - Weak (W.), Decision Process - References (R), Decision Process - Evidence (E.), Source - External Pages (E.P.), Description - Useful (U.), Description - Not Useful (N.U.), Condifence - High (H.), Confidence - Medium (m), Confidence - Low (L.), Time - Few more (F.M.) Each cell of the table is the result of Spearman correlation, and we use “*” to indicate when the p-value is below 0.05, therefore, the correlation is significant. For example in 51% of the cases, when an evidence is weak the participant needed more time to make a choice

### Perception Analysis - Think Aloud Experiment

All interviewees agree that Prospector report is useful to determine the fixing commit, for example, participant 6 said: *“I think it’s very helpful because it finds out what to look for, instead of doing a manual search.”*. Participant 4 made a reflection regarding the vulnerability description extracted by the NVD page which in some cases is not enough informative, and having Prospector retrieving the possible patch facilitates the task of searching the right fixing commit: *“[...] it is quite difficult to get the idea of the vulnerabilities on the description. Sometimes, the descriptions are high level, and they do not tell information about the specific fix. [...] I found that the commits tell more information than the CVE description”*. In addition, participant 1 suggested to better improve the way labels are presented:*“[...] what bothers me is really the way these labels are presented”*.

Regarding the time to perform the task, the participants stated that it depends on the difficulty of the vulnerability:*“Five minutes is fine for the easy cases, I think. But for the hard cases, it is definitely not enough. I would say this is the best answer.”* said participant 1.

All the participants agreed that the task to perform was clear and that they thought the overall experience was good.

#### Takeaway

The rules are informative and have a significant impact on the decisions made by senior participants when identifying the vulnerability patch. ML models could consider incorporating these features as strong candidates for explainability approaches.

## RQ3: Assessment with Junior Experts

### Demographics and Expertise - Junior Participants

A total of 90 Computer Science Master’s students participated in the first phase of the experiment which was collected over two years (2023 and 2024). We performed the second phase of the experiment with a small group of 19 students from University 2 in February 2024. Among them, 69 were enrolled in the course 1 of University 1, the others in the course 2 of University 2. As a population, this is consistent with several other studies (Naiakshina et al. 2017, 2018; Rong et al. 2012; Chong et al. 2021).

To validate their experience, we first looked at their Java expertise as collected during the background phase. The scale values ranged from *“No experience with Java”* to *“Expert, has used this language in several projects”*.

Both groups from either universities have written several programs in Java in hands-on labs or during short internships.

Most of the participants (41%) are somewhat familiar with the language and know the basics. Some participants (28%) are really familiar with the language and have at least used it for a project. A small percentage of the participants (10%) declared themselves experts as they used Java in several projects. Finally, 10% of the participants are slightly familiar with the language, and another small group (11%) does not have experience since they have never used the language.

As validation, we performed a small test to check whether the participants answered by chance when performing the experiment. We found that if the participants answered by chance, the mean of correct answers would be 0.5; however, on average the participants found 1.8 correct answers in total. Therefore, we consider our population a good sample for answering our research questions.

### Results

This section describes (1) the execution plan of the experiment, (2) the process adopted to select CVEs to perform the experiment with junior experts, (3) the experimental variables collected, (3) the demographics and expertise of the participants, and (4) and the results we obtained.

To answer RQ3 we performed an empirical experiment with master students as we wanted to study (1) which tool’s features are most informative to identify the fixing commit, (2) and how junior users perform when using Prospector. We report the results of our study in the following sections.

#### Phase 1 - Results

Table 11 shows the result we obtained from the first phase of the experiment. At the top of the table we counted the total number of students that found the right commit and did not find it for each of the three different. The row ’TOT’ shows that there is not much difference among the three different treatments. We used a Chi-square test to check for the statistical test and we obtained $\chi ^2(474, df=2)= 0.57, p = 0.75$, therefore there is no significance difference between the groups. This could be because 10 minutes is a quite reasonable time to have a look at most of the commits and have a similar rate of success.

**Table 11:** Results from the Juniors Evaluation

	Control	Rules	Relevance	TOT
Found	63	64	57	184
Not Found	95	94	101	290
TOT	158	158	158	474
	39.87%	40.51%	36.08%	38.82%

#### Phase 2 - Validation

The 95% C.I. of the probability of identifying the correct fixing commit is [0.62, 0.76]. Therefore, we consider this to be a significant result given the lack of experience of the participants.

#### Takeaway

When introduced to a new task, juniors and experts perform comparably. Moreover, presenting the top 10 commits for a specific vulnerability is sufficient to reduce the time required to identify the vulnerability patch, provided that the rules are sufficiently informative.

### Perception Analysis

#### Junior Evaluation - Part 1

The majority of the students stated that the time was sufficient to perform the task (79%), 20% of the participants said that the time was sometimes sufficient, sometimes not, in particular one participant commented *“The time should be increased since we have to look through several commits.”*. Only one participant stated that the time was not sufficient.

Most of the participants strongly agreed (9%) and agreed (48%) that the Prospector report is useful to identify the fixing commit, 33% of the participants neither agreed nor disagreed and few participants disagreed (4%) and strongly disagreed (2%). The participants strongly agreed (47%) and agreed (32%) that the task was clear: *“The instruction is sufficient and the task is clearly introduced.”*, 16% neither agree nor disagree, and only 5% disagreed or strongly disagreed.

Moreover, we asked an opinion whether the experiment was overall a good experience. More than half of the participants strongly agreed (13%) and agreed (46%) it was a good experience, one participant stated: *“I believe this exercise is very good and I believe that further examples using this tool could be helpful in a better understanding of vulnerability threats.”*, another said: * “This exercise is very funny, I think, it allows to highlight the usefulness of a tool. What is very interesting is that either in less than 5 minutes I am almost sure which commit is the fix, or either I would really need a lot more time to analyze the commits. It is very easy or very hard.”*. Some participants (34%) neither agreed nor disagreed, and few participants (7%) disagreed or strongly disagreed.

#### Junior Evaluation - Part 2

We asked the same perception questions for the second part of the experiment. Most of the participants strongly agreed (11%) and agreed (63%) that the Prospector report is useful to identify the fixing commit, 26% of the participants neither agreed nor disagreed. The participants strongly agreed (42%) and agreed (47%) that the task to perform was clear and 11% neither agreed nor disagreed.

Half of the participants (53%) said that time was sometimes sufficient, sometimes not, considering the number of participants (32%) who think time was sufficient. Finally, 16% said that the time was not sufficient.

Regarding the overall experience of the experiment, 63% of the participants agreed that the experience was good and 11% of them were strongly agreed. Some participants (26%) neither agreed nor disagreed.

#### Takeaway

The results indicate that the experimental design is effective. Most participants found the task clear, the allotted time sufficient, and the Prospector report helpful in identifying the vulnerability patch.

## Threats to Validity

### Dataset Size

A potential limitation of this study lies in the size of the dataset. A larger dataset would allow for more extensive evaluations and possibly the inclusion of additional baselines, such as deep learning models. However, constructing such datasets automatically would require using heuristic rules similar to those implemented in Prospector to link vulnerabilities to their patches. This would introduce a circular bias, as Prospector would naturally outperform all other methods by design. For this reason, we do not see a straightforward solution to this limitation without compromising the fairness of the evaluation.

### Population

The background and practices of the participants may impact the experiment’s results. Still, several studies with promising results have been conducted with students (Chong et al. 2021; Naiakshina et al. 2017, 2018; Rong et al. 2012). In fact, the study by Salman et al. (2015) compared the performances between students and professionals to investigate how well students represent professionals as experimental subjects. When approaching something new for the first time, the performance is similar for both subject groups. The time measurement might not exactly reflect the actual time participants spend on the tasks. We plan to investigate these limitations with further studies. Tahaei and Vaniea (2022) performed an experiment to explore programming skills, privacy and security attitudes, and self-efficacy in secure development of participants from a CS student mailing list and four crowd-sourcing platforms (Appen, Clickworker, MTurk, and Prolific). The results show that 89% of the CS students correctly answered all programming skill questions compared to 27% of the crowd-sourcing participants.

Finally, according to the 2021 developer survey of Stackoverflow^7^, more than 58K professional developers responded and for 49.34% the Bachelor’s degree is the highest education. A significant part (19.08%) is between 18 and 24 years old. These statistics are aligned with our population. However, we acknowledge that our population is not representative of different types of developer (e.g., those without a degree); more studies should be performed.

Providing more information about the characteristics of the junior assessors – such as their experience with version control systems, bug tracking tools, and security analysis – would have strengthened the background analysis. However, since our goal was to conduct an experiment involving participants without a strong security background, in order to assess whether Prospector can provide valuable insights even in the absence of security expertise, we considered basic proficiency in Java to be the minimum requirement for participation.

### Contamination by Results from Previous Year

For all the experiment iterations we used the same vulnerabilities dataset, except for the second phase, which was only one iteration. This choice might arise because of the contamination effect on participants in year N by the results of the previous year. However, since the experiment does not affect the grade of the students to pass the course, the students are not motivated to share the results between them. Moreover, when the students were asked to analyze the data of their fellows, we made available their data, but also the data from the previous year. Also, we did not observed a significant improvement of the results through the years; therefore, we think that the participants are not affected by the results from the previous year.

### Training Duration

Comparing to threat analysis and security requirements, our training session is comparatively short as their sessions last several hours (Wuyts et al. 2014; Scandariato et al. 2015) or even days (Tuma and Scandariato 2018).

However, in the domain of code review, Chong et al. (2021) performed the experiment after several weekly lectures with 60 minutes a week. On the opposite side of the spectrum, among the works cited, Tao et al. (2014) provide only a 10 minute tutorial. Several other works did some introduction, instruction or tutorial for their participants, but they do not mention the length of the session (Naiakshina et al. 2017, 2018; Cambronero et al. 2019; Zhang et al. 2022; Fry et al. 2012). Other works (Rong et al. 2012; Gonçalves et al. 2020) do not mention any training for their participants. Braz et al. (2022) mention training as one of their control variables, but did not actually perform any training, as their experiment was an online experiment.

### Ground Truth Selection

The analysis to reply to RQ1 was based on using Tracer as a ground truth since we did not have any other resource. This might have influenced the results obtained, however, we obtained results that perform similarly to the security experts defined common sense rules. Therefore we think that if by redefining the ground truth in a different way (at the moment the only option would be manually), we would obtain results that confirm what we already observed.

### Generalizability of the Results

The restricted selection of the CVEs may affect the generalizability of the results. In the future, more experiments with different CVEs, slicing algorithms, and code projects can be performed to further analyze and improve the generalizability of the results.

## Conclusion and Future Works

In this article, we present a study aimed at evaluating an alternative approach to machine learning models for identifying vulnerability patches. The proposed approach relies on expert-defined common-sense rules with power-law weights, implemented in the Prospector tool.

The first part of the study included a comparison between the expert-defined rules and four traditional ML techniques–linear regression, logistic regression, XGBoost, and LightGBM. This comparison was performed only as a *control study* to validate the soundness of the tool before conducting the human-centered evaluation, which represents the main contribution of this work. We observed that the ML models and the expert-defined rules achieved comparable results across standard metrics (precision, recall, accuracy, and HSS), suggesting that the rule-based approach is effective even in the absence of large training datasets. Moreover, the ranking of rule importance derived from Shapley values closely matched the weights assigned by experts, confirming that the heuristics encoded in Prospector are well aligned with the patterns learned by ML models.

The central goal of our study was to assess whether these expert-defined rules are informative and supportive for users when identifying the fixing commit using Prospector. Through seven think-aloud interviews with senior security experts, we observed that participants were faster and more confident in their decisions when commits matched highly informative rules such as CVE_ID_IN_MESSAGE or COMMIT_MENTIONED_IN_REFERENCE. In contrast, lower-ranked rules were associated with slower decisions and lower confidence, indicating that the rule set effectively influences human judgment and provides meaningful guidance.

Finally, we conducted a controlled experiment with master’s students to evaluate the experience of less experienced users when using Prospector for the first time. The results reported in Section 7 show that junior participants achieved performance comparable to senior experts when both groups were introduced to a new task. Furthermore, presenting the top-ranked commits suggested by Prospector significantly reduced the time needed to identify the fixing commit, especially when the matched rules were informative.

While our study is instantiated using Prospector, we view Prospector as a concrete example of a broader class of rule-based, explainable decision-support tools for vulnerability patch identification. The empirical findings are therefore not tied to a specific implementation, but rather to the design principles underlying such systems: the use of transparent, expert-defined rules; explicit weighting schemes; and ranked recommendations presented to human analysts.

Our results suggest that (i) expert-defined rules can capture patterns comparable to those learned by ML models, (ii) informative and well-ranked rules meaningfully influence human decision-making, and (iii) presenting a limited set of top-ranked candidates can reduce analysis time without degrading performance. We expect these takeaways to generalize to similar rule-based or hybrid decision-support systems in which interpretability and alignment with human reasoning are central design goals.

Future work could extend this research through additional interviews with expert users (under a newly approved ethics protocol) to refine the guidelines for using expert-defined rules and to further analyze differences between experienced and novice users in real-world conditions.

Another interesting direction for future work is the application of the methodology to evaluate deep learning or other machine learning tools once they are enabled with explainability features. The ranking and the explained features could then be used to define the human experiment as we have done (with highlighted and not highlighted explained features). Unfortunately, such experiments are scientifically undermined by the current small datasets that are unsuitable for true DL and LLM models (Kaplan et al. 2020) and, most importantly, by the fact that they are all build by using either Prospector itself or Prospector top rule (CVE in the commit) to build the ground truth for training.

On a similar note, one can directly adapt our artifact and apply our methodology to a human evaluation of (silent) patch localizations performed by LLMs. In such scenarios, human users would be either shown the commits in the order generated by the LLM (but no explanation) or the temporal ordering of the commits and the corresponding explanations. To this extent it would be essential to use a novel dataset created after the release of the tested LLMs.

## Data Availability

The replication package is available on Zenodo. The reviewers can directly access the raw data at this link: https://doi.org/10.5281/zenodo.17471849. It contains the raw data of the participant’s responses, the reviewers may check the results. The tool Prospector is available on GitHub at this link: https://github.com/sap/project-kb.

## Footnotes

1 https://nvd.nist.gov

2 https://shap.readthedocs.io/en/latest/index.html

3 https://www.itl.nist.gov/div898/handbook/prc/section2/prc241.htm

4 https://www.secura.com/services/people/training-courses/secure-programming-training

5 https://github.com/SAP/project-kb

6 https://docs.scipy.org/doc/scipy/reference/generated/scipy.stats.spearmanr.html

7 https://insights.stackoverflow.com/survey/2021

## Declarations

### Employment

S.E. Ponta and A. Sabetta are employees of SAP.
